# Fluorescence Lifetime Nanoscopy of Liposomal Irinotecan Onivyde: From Manufacturing to Intracellular Processing

**DOI:** 10.1021/acsabm.3c00478

**Published:** 2023-09-12

**Authors:** Mario Bernardi, Giovanni Signore, Aldo Moscardini, Licia Anna Pugliese, Luca Pesce, Fabio Beltram, Francesco Cardarelli

**Affiliations:** † Scuola Normale Superiore, Laboratorio NEST, Piazza San Silvestro 12, 56127 Pisa, Italy; ‡ Biochemistry Unit, Department of Biology, University of Pisa, via San Zeno 51, 56123 Pisa, Italy; § Institute of Clinical Physiology, National Research Council, 56124 Pisa, Italy; ∥ NEST, Istituto Nanoscienze-CNR, Piazza S. Silvestro, 12, I-56127 Pisa, Italy

**Keywords:** onivyde, irinotecan, SN-38, phasor FLIM, fluorescence, INS-1E cells

## Abstract

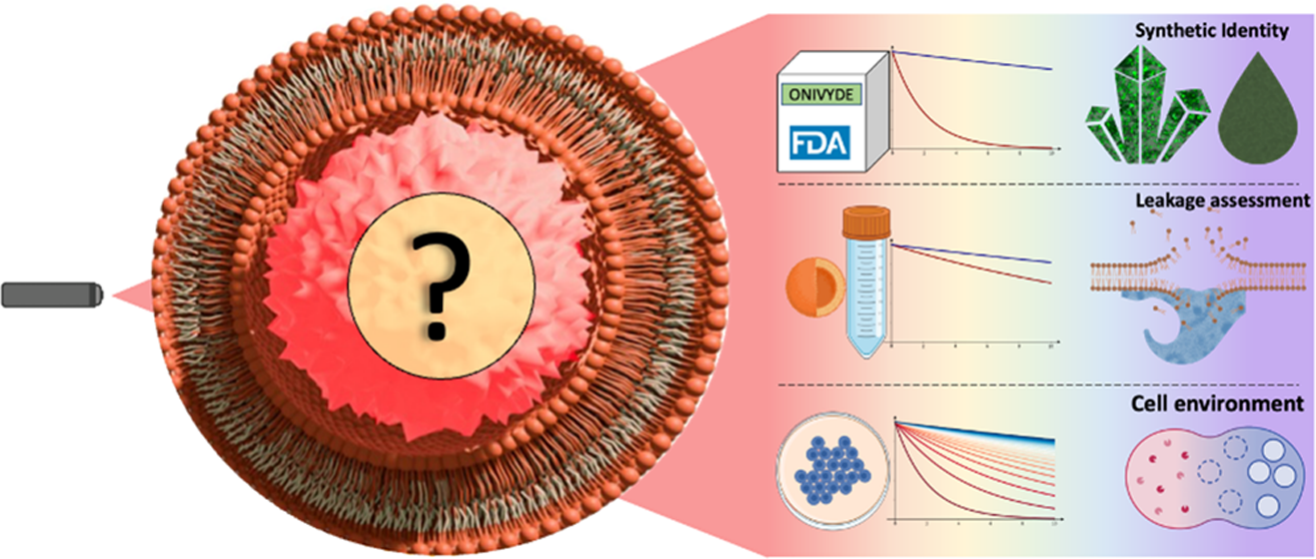

Onivyde was approved by the Food and Drug Administration (FDA) in 2015 for the treatment of solid tumors, including metastatic pancreatic cancer. It is designed to encapsulate irinotecan at high concentration, increase its blood-circulation lifetime, and deliver it to cells where it is enzymatically converted into SN-38, a metabolite with 100- to 1000-fold higher anticancer activity. Despite a rewarding clinical path, little is known about the physical state of encapsulated irinotecan within Onivyde and how this synthetic identity changes throughout the process from manufacturing to intracellular processing. Herein, we exploit irinotecan intrinsic fluorescence and fluorescence lifetime imaging microscopy (FLIM) to selectively probe the supramolecular organization of the drug. FLIM analysis on the manufacturer’s formulation reveals the presence of two coexisting physical states within Onivyde liposomes: (i) gelated/precipitated irinotecan and (ii) liposome-membrane-associated irinotecan, the presence of which is not inferable from the manufacturer’s indications. FLIM in combination with high-performance liquid chromatography (HPLC) and a membrane-impermeable dynamic quencher of irinotecan reveals rapid (within minutes) and complete chemical dissolution of the gelated/precipitated phase upon Onivyde dilution in standard cell-culturing medium with extensive leakage of the prodrug from liposomes. Indeed, confocal imaging and cell-proliferation assays show that encapsulated and nonencapsulated irinotecan formulations are similar in terms of cell-uptake mechanism and cell-division inhibition. Finally, 2-channel FLIM analysis discriminates the signature of irinotecan from that of its red-shifted SN-38 metabolite, demonstrating the appearance of the latter as a result of Onivyde intracellular processing. The findings presented in this study offer fresh insights into the synthetic identity of Onivyde and its transformation from production to in vitro administration. Moreover, these results serve as another validation of the effectiveness of FLIM analysis in elucidating the supramolecular organization of encapsulated fluorescent drugs. This research underscores the importance of leveraging advanced imaging techniques to deepen our understanding of drug formulations and optimize their performance in delivery applications.

## Introduction

Onivyde consists of ∼110 nm diameter liposomes with 2000 Da segments of poly(ethylene glycol) (PEG) engrafted onto the lipidic surface and loaded with a high concentration (i.e., 4.3 mg/mL) of sucrose octasulfate irinotecan in a gelated/precipitated state.^1^ Irinotecan is a camptothecin-derived prodrug that is subject to metabolic conversion by intracellular carboxylesterases^2^ into SN-38, a metabolite with 100- to 1000-fold higher anticancer activity.^3^ Irinotecan encapsulation by lipids attenuates prodrug poor water solubility, increases its stability and overall lifetime in circulation (e.g., by preventing early conversion into SN-38), and favors its accumulation at the tumor site by a well-known enhanced permeability and retention (EPR) effect.^4^ In 2015, the FDA approved the use of Onivyde for the treatment of multiple solid tumors, in particular metastatic pancreatic cancer (MPC).^5^ Indeed, preclinical and clinical tests demonstrated the superior efficacy of Onivyde in prolonging the overall survival of patients with MPC, compared to the isolated drug.^6−9^ In addition, the combined use of Onivyde and 5-fluorouracil/leucovorin (5-FU/LV) has emerged as a valuable treatment option for second-line MPC patients and is being evaluated by ongoing phase-III clinical trials (NAPOLI-3; NCT04083235) as a first-line treatment option.^10^ In spite of such a rewarding clinical track record, surprisingly little is known about the supramolecular organization of liposome-carried irinotecan from manufacturing (i.e., synthetic identity) to intracellular processing and the final fate (i.e., biological identity). This limited understanding significantly hampers one’s capacity to effectively enhance the performance of encapsulated irinotecan in delivery applications and propose novel formulations through rational design. In line with this objective, we have recently introduced an innovative strategy that capitalizes on the inherent fluorescence of a drug coupled with advanced fluorescence lifetime imaging microscopy (FLIM) techniques. This approach enables us to selectively investigate the nanoscale organization of the drug, yielding valuable insights into its behavior and distribution.^11^ FLIM analysis of irinotecan intrinsic fluorescence revealed the presence of two coexisting physical states of the prodrug within liposomes in the manufactured solution: (i) gelated/precipitated irinotecan and (ii) irinotecan associated with the liposome membrane. It is noteworthy that the presence of the latter state is not discernible from the indications alone. Moreover, FLIM used in combination with high-performance liquid chromatography (HPLC) and with a membrane-impermeable dynamic quencher of the irinotecan signal revealed that irinotecan supramolecular organization changes dramatically upon Onivyde dilution into a typical cell-culturing medium, with rapid and complete dissolution of the gelated/precipitated phase and its conversion into free-in-solution irinotecan. In line with its marked instability upon dilution in cell-culturing medium, Onivyde resulted barely distinguishable from nonencapsulated irinotecan in terms of both cell-uptake mechanism and cell-division inhibition when tested by means of confocal imaging and cell-proliferation assays, respectively. Finally, 2-channel FLIM analysis allowed us to discriminate the signature of irinotecan from that of the red-shifted SN-38 metabolite, demonstrating the appearance of this latter as a result of Onivyde intracellular processing. Reported results, besides validating FLIM analysis as a tool to complement standard methods for drug investigation, shed light onto Onivyde synthetic identity and its evolution from production to in vitro administration.

## Materials and Methods

### Materials

Liposomal irinotecan Onivyde was donated to Scuola Normale Superiore by the Medical Affair Department of Servier Italia S.p.A. One 10 mL vial of sample contains 43 mg of irinotecan anhydrous free base in the form of irinotecan sucrosofate salt in a pegylated liposomal formulation. The liposomal vesicle is composed of 1,2-distearoyl-*sn*-glycero-3- phosphocholine (DSPC) 6.81 mg/mL (1:1.6), cholesterol 2.22 mg/mL (1:0.5), and methoxy-terminated poly(ethylene glycol) (*M*_W_ 2000)-distearoylphosphatidyl ethanolamine (MPEG-2000-DSPE) 0.12 mg/mL (1:0.03). Each mL also contains 2-[4-(2-hydroxyethyl) piperazin-1-yl] ethanesulfonic acid (HEPES) as a buffer 4.05 mg/mL and sodium chloride as an isotonicity reagent 8.42 mg/mL. Irinotecan hydrochloride (powder), purchased from Sigma-Aldrich (Milan, Italy), and Onivyde were both stored at 4 °C in compliance with the datasheet. The melting temperature of DSPC is reported to be 54 °C,^12^ whereas MPEG-DSPE gel melting temperature is reported to be higher than 74 °C.^13^ In this study, we evaluated the impact of pH variation on the characteristic lifetimes of irinotecan and its metabolite SN-38, purchased from TCI Europe N.V. (Zwijndrecht, Belgium). The pH range studied was from 2.0 to 12.0, and the buffer used was PBS due to its compatibility with living cells and broad buffering capacity. To simplify the methodology, we opted to use PBS rather than more complex buffer mixtures, despite their higher buffering capacity. Nine PBS solutions were prepared with the desired pH, starting from stock solutions of irinotecan and SN-38 in DMSO. 1 mM solutions in PBS were then prepared for each pH point, and the final solutions were stirred to maintain the pH control.

### Sample Preparation

To prepare precipitated irinotecan, following Ipsen Biopharm Ltd. patent,^14^ we dissolved 1.64 mg of irinotecan hydrochloride in DI water pH = 5.0 and subsequently heated the solution in a thermomixer at 65 °C for 30 min. Upon complete dissolution, we introduced 5 μL of a solution containing 3.68 M ammonium sulfate to replicate the precipitation of irinotecan according to stoichiometric reaction eq 1.
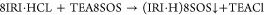
1

We replaced the reagent triethylammonium sucrosofate with ammonium sulfate by simply acknowledging that sucrosofate would account for 8 sulfate groups. The solution was stored overnight in an Eppendorf AG (Hamburg, Germany) black glass bottom 96-well plate to rest until observing a gel-like precipitated irinotecan phase. To mechanically destroy Onivyde, samples were seeded on a glass Petri dish and then spin-coated for 1 min at 5000 rpm. The aqueous solution is naturally lost during the procedure. Irinotecan precipitate or membrane patches adhere directly on the glass. Irinotecan hydrochloride solubility in water is very scarce if not enhanced by acidified solutions. To isolate the free phase of irinotecan dissolved in solution, we relied on dissolution in dimethyl sulfoxide (DMSO), purchased from Sigma-Aldrich (Milan, Italy), as it yields a 50 mg/mL solubility. A 1 mM DMSO stock, stored at 4 °C, was used to prepare diluted solution in various buffers (i.e., water, saline solution for intravenous injection, and cell-culturing medium).

### Cell Culture

Insulinoma 1E (INS-1E) cells were a kind gift from Professor C. Wollheim from the University of Geneva. These cells were kept in a climate-controlled incubator set to 37 °C and 5% CO_2_, where they were grown in RPMI 1640 medium containing 11.1 mmol/L d-glucose, 10 mmol/L HEPES, 2 mmol/L l-glutamine, 100 U/mL penicillin–streptomycin, 1 mmol/L sodium pyruvate, and 50 μmol/L β-mercaptoethanol. To conduct lifetime experiments, the cells were allowed to grow until they reached 70% confluence on sterilized microscopy-compatible dishes (IbiTreat μ-Dish 35 mm, Ibidi) for a period of 24–48 h. Then, the cells were exposed to either irinotecan or Onivyde both diluted in complete medium. To serve as a control, the cells were simply refreshed with a fresh batch of the complete medium.

### Fluorescence-Intensity Measurements

Quenching measurements were performed on a Cary Eclipse fluorescence spectrometer (Agilent) by monitoring the fluorescence of a 1 μM solution of Onivyde in various media, well within the fluorescence linear range of irinotecan.^15^ The solution was subsequently diluted in a buffer containing potassium iodide (KI) until the concentration of KI reached 300 mM. To evaluate the quenching effect, a control solution containing Onivyde was also prepared and diluted in the same volumetric ratio as the experimental solution but in the absence of KI. The fluorescence of the experimental solution containing KI at various concentrations (0–300 mM) was then compared to the control solution at corresponding dilutions to assess the quenching effect, according to eq 2.
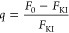
2

To investigate colocalization of Onivyde or irinotecan with lysosomes in INS-1E cells, we used LysoTracker Deep Red (Thermo Fisher) as a far-red dye to avoid crosstalk with irinotecan. Custom-made Python 3.6 routines were employed for data analysis, incorporating Otsu’s method of Otsu et al. to calculate a threshold in both acquisition channels. Co-occurrence measures, including the Pearson coefficient (*r*) and Manders coefficients (*M*_1_ and *M*_2_), were calculated to determine the percentage of the signal overlap between channels, as per eq 3.a–3.c.
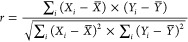
3a
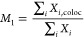
3b
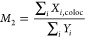
3c

### Proliferation Assay

To perform proliferation assays, cells were treated with Onivyde, irinotecan, or SN-38 for 24 h. Control and treated cells were then fixed with 4% PFA in PBS for 10 min at RT (room temperature) and washed 3 times with PBS, 5 min each. After fixation, cells were permeabilized with PBS + 0.1% triton X-100 (PBST) for 10 min at RT, washed 3 times with PBS, and then blocked with 1% bovine serum albumin (BSA) in 0.1% PBS TWEEN for 30–45 min at RT. The samples were incubated with the primary antibody for Ki67 (rabbit polyclonal, 15,580, abcam), (diluted 1:100 in 0.1% PBS TWEEN) overnight at 4 °C. Then, after 3 washes with PBS for 5 min each, the specimens were incubated with secondary antibody antirabbit Alexa Fluor 647 (donkey antirabbit, A31573, Thermo Fisher Scientific) diluted 1:100 in 0.1% PBS TWEEN and 1% BSA for 1 h at RT. The stained samples were then washed 3 times with PBS (5 min each) and then washed with 1 μg/mL DAPI in PBS for 10 min. Fixed samples stained for Ki67 were acquired with an inverted Zeiss LSM 800 confocal microscope (Jena, Germany). The acquisition was performed by illuminating the sample with 353 and 653 nm lasers using a 40 × /NA 1.3 oil-immersion objective. DAPI and Alexa Fluor 647 fluorescence were collected between 410 and 617 nm and 645 and 700 nm, respectively, with GaAsP detectors. The pinhole aperture was set at 44 μm.

### FLIM Measurements

Before each FLIM measurement, a drop of approx. 20 μL of Onivyde was diluted 50× in 980 μL of saline as per the intravenous administration protocol. The solution was poured onto the glass of a WillCo plate, without any further dilution. For what concerns the free drug, the 1 mM irinotecan stock solution in DMSO was diluted in different buffers prior to FLIM at a final concentration of ∼10 μM. Irinotecan precipitate and spin-coated liposomes were obtained on the glass of a WillCo plate and black glass bottom 96-well plate, respectively, as described above. No aqueous solution was added prior to FLIM to avoid any possible drug resuspension. FLIM measurements were performed by an Olympus FVMPE-RS microscope coupled with a two-photon Ti/sapphire laser with 80 MHz repetition rate (MaiTai HP, SpectraPhysics) and a FLIMbox system for lifetime acquisition (ISS, Urbana–Champaign). Onivyde and irinotecan were excited at 760 nm, and the emission was collected by using a 30× planApo silicon immersion objective (NA = 1.0) in the 380–570 nm range. Calibration of the ISS FLIMbox system was performed by measuring the known monoexponential lifetime decay of fluorescein at pH = 11.0 (i.e., 4.0 ns upon excitation at 760 nm, collection range: 570–680 nm). To prepare the calibration sample, a stock of 100 mmol/L fluorescein solution in EtOH was prepared and diluted in NaOH at pH 11.0 for each calibration measurement. For each measurement, a 512 × 512 pixel image of FLIM data was collected until 30 frames were acquired. Figure S1 eliminates the possibility that the lifetime value is the result of second harmonic generation (SHG). To rule out SHG, we split the signal through a dichroic filter and gathered the signal in two different intervals: 380–470 and 470–570 nm.

### Phasor Analysis of FLIM Data

The phasor analysis of experimental lifetime acquisitions was performed by using custom dedicated routines implemented in Python 3.6. Technically, for each pixel in the image, the fluorescence decay measured in the time domain is mapped onto the so-called “phasor” plot, where a phasor is described by two coordinates: the real and imaginary parts of the Fourier transform of the fluorescence lifetime decay, calculated at the angular repetition frequency of the measurement. Thus, pixels with similar decay curves show similar coordinates in the phasor plot; also, pixels containing a combination of two (or more) distinct lifetime decays will be mapped according to the weighted linear combination of these contributions (see Figure S2). Equations 4.a,4.b describe the computation of the coordinates considering *n* and ω, harmonic and angular frequency, respectively.
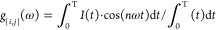
4a
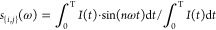
4b

In the frequency domain for each pixel, one can rely on the modulation *m*_*i,j*_ and phase shift ϕ_*i,j*_ of the signal as reported in eqs 5.a,5.b
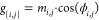
5a
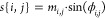
5b

The phasors lie within the semicircle, which goes by the name of a universal circle, centered at (1/2,0) with a radius 1/2 and positive *x*, where the zero lifetime is located at (1,0) and the infinite lifetime at (0,0). Indeed, by taking the Fourier transformation of a measured decay curve, the lifetime can be estimated relying solely on the position of the phasor inside the universal circle. The distribution of phasor points originating from FLIM measurements is found on the universal circle for monoexponential decays or within the universal circle for multiexponential decays. In the case of a monoexponential decay, the intensity can be expressed according to eq 6.a, whereas multiexponential decay intensity can be expressed according to eq 6.b, where subscripts f, b, and p indicate irinotecan in free, membrane-associated, and gelated/precipitated forms, respectively.
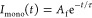
6a
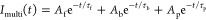
6b

If two molecular species are coexisting in the same pixel, for instance, all of the possible weighting combinations of the two molecular species give phasors distributed along a straight line joining the characteristic phasors of the two pure species; in the case of three molecular species, the possible combinations of the system fill a triangle where the vertices correspond again to the characteristic phasors of the pure species.^16−19^ As shown in Figure S2, given an experimental phasor that is the combination of two (or more) species and the phasors of the isolated pure components, a graphical solution can be derived as described previously by some of us.^11^ The graphical solution can be mathematically described in terms of intensity fraction F and characteristic lifetime τ of each species as per eq 7.a, whereas the molar fraction of each species can be computed as per eq 7.b as a function of the molar extinction coefficient ε and quantum yield (QY). Equation 7.c is a simplified version of (7.b), based on well-documented considerations reported in the literature.^11,20−22^
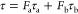
7a
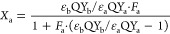
7b
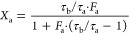
7c

We calculated the uncertainty in the final measurements as the standard deviation for a single measurement. However, systematic errors may arise due to the approximations used in the analysis. For example, our analysis assumed that the absorption coefficient of the fluorophore is the same for all physical states of irinotecan but only accounted for a weaker nonradiative decay in the membrane form. Moreover, we could not measure the QY of precipitated irinotecan since the precipitation protocol involves the use of a glass bottom 96-well plate, from where it is not possible to retrieve a “pure” sample of precipitated irinotecan. The presence of subpopulations in the phasor plot was investigated by using the Kolmogorov–Smirnov and Shapiro tests on lifetime histograms. To achieve successful segmentation, we used the deep learning-based method Cellpose.^23^

### HPLC Release Assay

The methodology employed for the Onivyde release assay is described as follows. Initially, 100 μL of Onivyde was diluted in 1.0 mL of buffer solution and introduced into a 1 kDa molecular weight cutoff dialysis membrane. The membrane was sealed and placed in a 50 mL Falcon tube containing 25 mL of buffer solution prewarmed to 37 °C using a thermomixer. The release assay was conducted with gentle shaking at 300 rpm at a constant temperature of 37 °C. At predetermined time intervals, 50 μL of external buffer solution was collected and subjected to mass analysis (3200 QTrap ABSciex) using an HPLC Nexera instruments system (Shimadzu). For the chromatographic separation, a Kinetex EVO C18 column (Phenomenex) was used. The aqueous phase consisted of a 15 mM ammonium acetate solution at pH 7.0 (solvent A), and the organic phase (solvent B) consisted of MeOH/Aqueous solution (95:5). The analyses were performed under isocratic conditions using 55% of solvent B with a flow rate of 0.5 mL/min. The detection using mass spectrometry was performed in positive mode by using the following optimized parameters: ion source voltage 5000 V, source temperature 100 °C, ion source gas 20 L/min, curtain gas 10 L/min, declustering potential 60 V, and collision energy 40 V. Data gaining was obtained resorting to the multiple reaction monitoring (MRM) technique for irinotecan (587.4/502.3 *m*/*z*) and SN-38 (393.2/349.3 *m*/*z*). To initially quantify irinotecan in Onivyde, 20 μL of Onivyde was dissolved in a mixture containing 550 μL of methanol, 380 μL of RPMI, and 320 μL of DMSO. By such a preliminary procedure, we obtained a 4.5 ± 0.2 mg/mL concentration, in keeping with the one declared by the vendor (4.3 mg/mL). Calibration curves were generated by employing MultiQuant software.

## Results and Discussion

### FLIM Analysis of Irinotecan Supramolecular Organization within Onivyde

**Figure 1:**
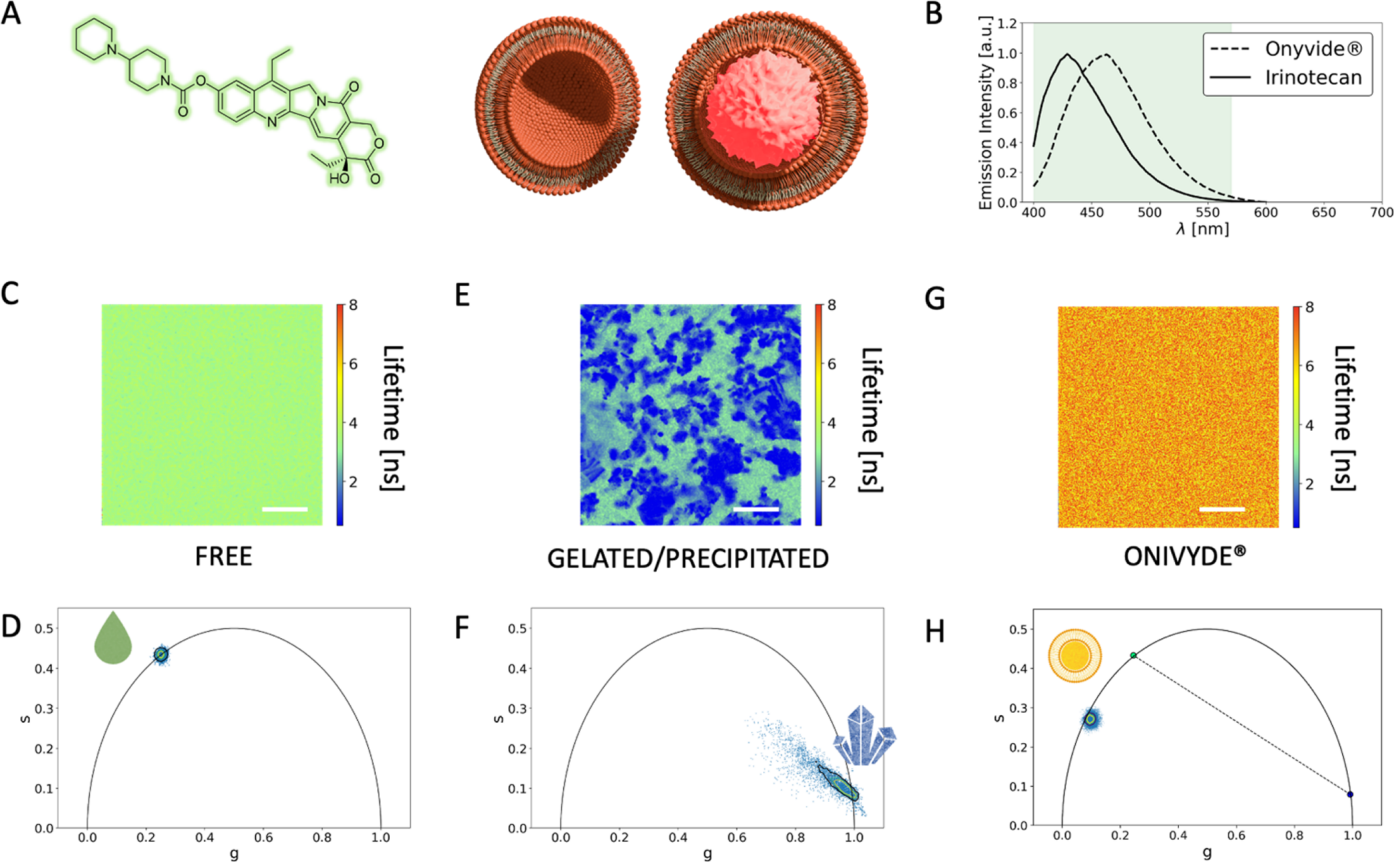
FLIM analysis of Onivyde synthetic identity. (A) Chemical structure of irinotecan (left) together with a schematic representation of its encapsulated form in a gelated/precipitated phase, as proposed by the manufacturer (right). (B) Fluorescence emission spectra of free-in-solution irinotecan (solid black line) and Onivyde (dashed black line). Shaded green areas enclose the range of wavelengths used to collect both signals. (C) Representative FLIM of free irinotecan in aqueous solution (lifetimes are color-coded according to the LUT on the right). (D) Phasor-plot representation of lifetime data from irinotecan in aqueous solution. (E) Representative FLIM of gelated/precipitated irinotecan. (F) Phasor-plot representation of lifetime data from gelated/precipitated irinotecan. (G) Representative FLIM of Onivyde in the manufacturer’s solution. (H) Phasor-plot representation of lifetime data from Onivyde in the manufacturer’s solution. The segment connecting the positions of free and gelated/precipitated irinotecan is represented as a reference (dashed line). Scale bars: 20 μm.

**Figure 2:**
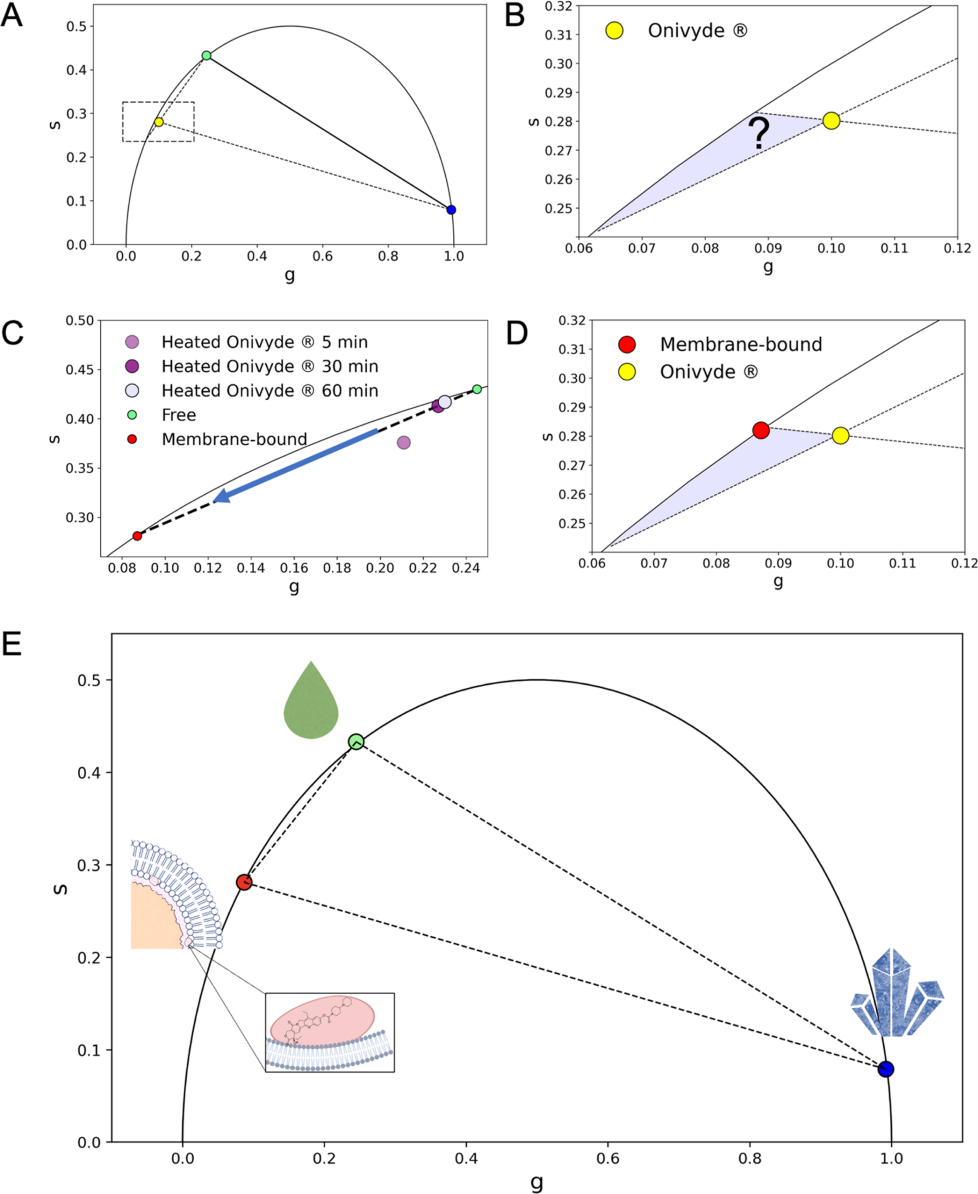
Third-species hypothesis to explain Onivyde lifetime data: experimental validation. (A) On the basis of the manufacturer’s indications, two pure species were measured, i.e., free-in-solution irinotecan (green dot) and gelated/precipitated irinotecan (blue dot). Experimental data from Onivyde (yellow dot) do not lie on the expected segment. (B) Detail of the phasor plot in panel (A) to better highlight the area of the plot where the putative third species is expected to lie. (C) Melting of gelated/precipitated irinotecan precipitate carried out at 90 °C for a maximum of 60 min (light violet dot); linear fitting across free-in-solution irinotecan and Onivyde heated at 90 °C for 60 min is used to identify the putative third species (red dot). (D) Same detail as in panel (B) but including the estimated position of the third species (red dot). (E) Phasor plot with the three pure species identified: free, gelated/precipitated, and membrane-associated irinotecan.

The irinotecan chemical structure is reported in Figure 1A. As previously shown^24,25^ and confirmed here, free-in-solution irinotecan has a detectable fluorescence spectrum peaked at around 430 nm. This fluorescence profile undergoes noticeable modifications (i.e., ∼40 nm red-shifted) upon liposomal encapsulation (Figure 1B). This in turn suggests that the liposome active loading process leads to a significant alteration in the nanoscale organization of the drug itself. Indeed, according to the manufacturer’s certificate of analysis,^1^ Onivyde liposomes contain most of the irinotecan molecules (∼95%) in a gelated/precipitated physical state (as a sucrose octasulfate salt) and only the remaining minor fraction (∼5%) as nonencapsulated molecules, i.e., irinotecan freely diffusing in solution. To probe the supramolecular organization of the drug, we used a recently validated experimental strategy based on fluorescence lifetime imaging microscopy (FLIM) and the phasor approach to FLIM data^11,26^ (see also the Materials and Methods section for further technical details). We started by measuring the phasor-FLIM signature of the two pure species expected according to the manufacturer’s indications: free and gelated/precipitated irinotecan. Irinotecan dissolved in aqueous solution (Figure 1C) yielded a characteristic monoexponential lifetime at around ∼3.4 ns, lying, as expected, on the “universal semicircle” in the phasor plot of Figure 1D (see also Table 1). To obtain irinotecan in a nearly pure gelated/precipitated physical state, we replicated the procedures described in the patent owned by Ipsen Biopharm Ltd.^14^ In brief, a water solution containing 1.64 mg of irinotecan hydrochloride at pH 5.0 was heated in a thermomixer at 65 °C for 30 min; then, 5 μL of a 3.68 M ammonium sulfate was added to initiate irinotecan precipitation. Micron-sized clusters of gelated/precipitated irinotecan were recovered on the glass, immersed in a solution of uniformly dispersed irinotecan (Figure 1E). By means of the high spatial resolution of confocal microscopy, the characteristic lifetime of the gelated/precipitated clusters could be isolated from the lifetime of monodispersed irinotecan. As reported in Figure 1F, the gelated/precipitated physical state is characterized by a nearly monoexponential lifetime centered at approx. 0.2 ns on the universal semicircle (see also Table 1). To validate these results, we performed a control experiment in which pristine Onivyde liposomes were spin-coated on a glass surface. This procedure mechanically destroyed the liposomal particles while recovering part of the material on the glass. Of note, phasor-FLIM analysis of the signal associated with the recovered material yielded a highly reproducible, nearly monoexponential lifetime at ∼0.2 ns (Figure S3, see also ref (11) for more details), thus matching the result from custom-made gelated/precipitated species. As a result, one would anticipate that all potential combinations of free and gelated/precipitated irinotecan, including Onivyde, would lie along the segment connecting the two pure species in the phasor plot (shown as a dotted black line in Figure 1H). However, the measured Onivyde phasors deviate from this expected segment (Figure 2A). In order to rationalize the experimental lifetime of Onivyde, at least one-third of species must be present in the mixture, with a characteristic lifetime signature located in the phasor-plot region highlighted in light purple in Figure 2B.

**Table 1:** All Values Are Expressed as Mean ± SD; *N* is the Number of Triplicate Independent Experiments; *Value Inferred by Exploiting the Heating Experiment (Described in Figure 2D)

		fractional intensity			
sample	lifetime [ns]	precipitated	free in solution	membrane-associated	*N*
free irinotecan	3.42 ± 0.02		100		9
gelated/precipitated irinotecan	0.21 ± 0.07	100			5
membrane-associated irinotecan	6.55 ± 0.46*			100	4
onivyde in buffer	6.37 ± 0.13	2.95 ± 0.09		97.05 ± 0.09	5
onivyde in saline solution	5.75 ± 0.10	1.00 ± 0.559	23.04 ± 2.60	75.96 ± 2.31	5
onivyde in saline solution (90 min)	5.60 ± 0.07	1.16 ± 0.07	26.51 ± 0.77	72.33 ± 1.71	3
onivyde in cell medium (5 min)	5.76 ± 0.12	0.18 ± 0.07	23.55 ± 1.82	76.27 ± 1.82	5
onivyde in cell medium (120 min)	4.04 ± 0.05		82.81 ± 0.03	17.19 ± 0.03	3

The third-species hypothesis was experimentally tested by selectively removing one of the two known species (i.e., gelated/precipitated irinotecan) from the liposomal formulation. In fact, as reported in the control experiment in Figure S3, increasing the temperature above ∼70 °C is sufficient to completely dissolve the gelated/precipitated phase into free irinotecan. If this protocol is applied to Onivyde, however, the phasor-FLIM signature of the sample never reaches that of free irinotecan (Figure 2C), maintaining a multiexponential nature that confirms the presence of at least one additional species. The position of this latter was inferred by fitting the data of Figure 2C and resulted in a monoexponential lifetime (Figure 2D) of about 6.55 ± 0.46 ns (see also Table 1). The strong similarities between encapsulated irinotecan and encapsulated doxorubicin (i.e., Doxil) in terms of both lipid composition and drug active loading procedures^28^ prompted us to speculate that the third species in Onivyde could correspond to a fraction of irinotecan molecules interacting with the liposome membrane. Worthy of note, the three-species reference system (Figure 2E) highlights the absence of free-in-solution irinotecan in the manufacturer’s formulation, a finding not entirely surprising based on irinotecan poor water solubility (approx. 0.5 mg/mL in a 1:1 solution of DMSO/PBS at pH = 7.2).

### FLIM Unveils Irinotecan Leakage upon Onivyde Dilution

**Figure 3:**
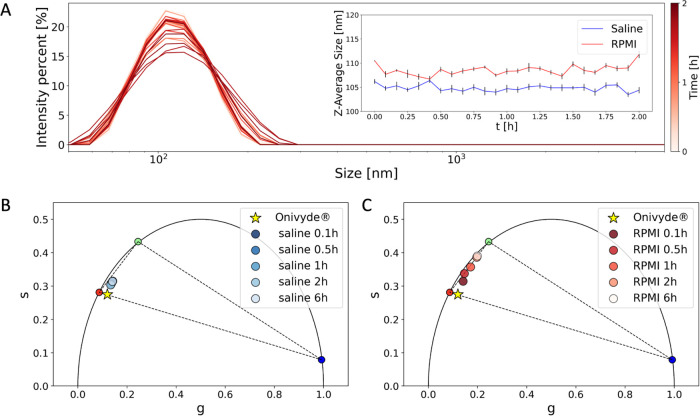
Synthetic identity of Onivyde changes upon dilution. (A) DLS measurements were performed to evaluate the characteristic size of Onivyde liposomes upon dilution and were repeated at intervals of 5 min between 0 h (e.g., light-red curve) and 2 h (e.g., dark-red curve). Two test solutions were probed: saline and the RPMI cell-culturing medium. As shown in the inset, minor differences in terms of liposome average size were reported in saline solution (blue) compared to RPMI medium (red) but were almost invariable over time. (B) Onivyde characteristic lifetimes in saline solution, monitored in the 0–6 h time frame after dilution. (C) Onivyde characteristic lifetimes in RPMI medium, monitored in the 0–6 h time frame after dilution.

**Figure 4:**
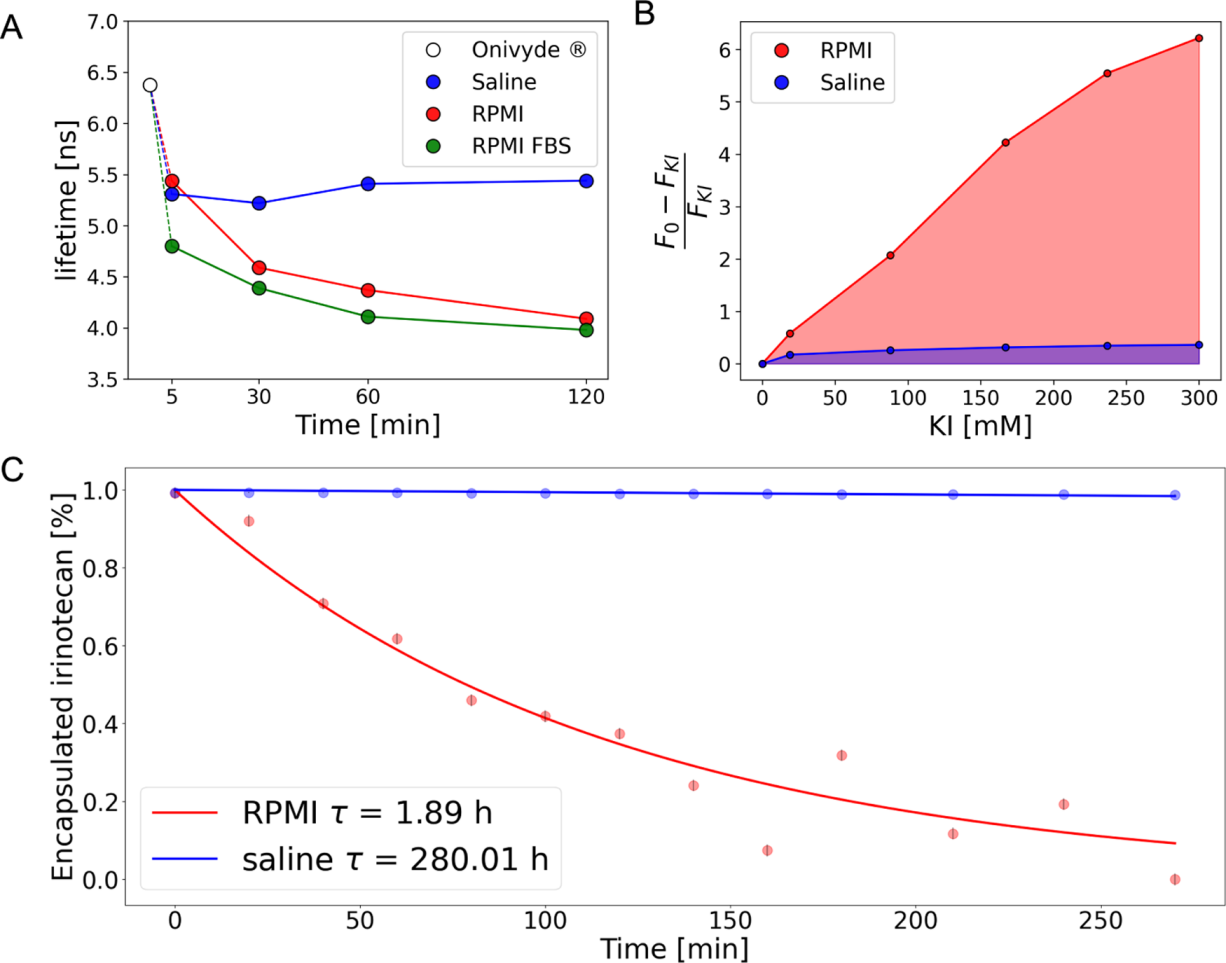
Irinotecan leakage analysis. (A) Average fluorescence lifetime of Onivyde over time upon dilution in saline solution (blue dots), RPMI medium (red dots), and FBS-enriched RPMI medium (green dots), measured at 37 °C between 5 min and 2 h (*N* = 3). (B) Residual irinotecan fluorescence as a function of increasing concentrations of KI in saline solution (blue) and RPMI medium at 37 °C (red). (C) HPLC-based irinotecan release assay conducted on Onivyde diluted in saline solution (blue) and RPMI medium (red) (*N* = 3). Interpolation of data points affords a quantitative estimate of the characteristic time of irinotecan leakage from liposomes into solution.

Simple algebraic calculations can be used to derive the fractional-intensity contributions of gelated/precipitated irinotecan (∼3%) and membrane-associated irinotecan (∼97%). However, these will not coincide with the actual molar fractions unless the distinct pure species had the same brightness. Still, the fractional-intensity framework can be used to monitor any variation of the Onivyde synthetic identity under different experimental conditions. We started by performing two dilution experiments of Onivyde, from manufacturer’s buffer into either the saline solution used for intravenous injection (i.e., 0.9% NaCl^1^) or the classical RPMI cell-culturing medium (with or without serum proteins). In both cases, Onivyde liposomes did not show any variation in size over time after the dilution step, as probed by dynamic light scattering (DLS) analysis (Figure 3A). This in turn implies that dilution is not associated with major liposome mechanical stress, i.e., liposome rupture and/or aggregation. By contrast, as reported in Table 1, the two dilution experiments could be clearly distinguished by FLIM analysis: indeed, while the fractional-intensity values of the pure species only slightly changed upon Onivyde dilution in 0.9% NaCl saline solution (Figure 3B), marked variations over time were detected upon Onivyde dilution in RPMI (Figure 3C). As shown in Figure 3C, a steady-state fractional-intensity distribution was reached within approx. 120 min, with a characteristic lifetime lying along the segment connecting the membrane-associated and the free-drug species. This result indicates that the gelated/precipitated phase of irinotecan completely dissolves upon Onivyde dilution in the cell-culturing medium. We observed an intriguing phenomenon in the initial phase of this experimental process, which involves a distinct temporal evolution, likely connected to the dilution step. Indeed, there is minimal disparity in the mechanical effect observed when diluting in saline or RPMI. However, the introduction of FBS into the cellular medium appears to trigger a more pronounced evolutionary phenomenon (see Table 2 and Figure 4A). Over time, it becomes evident that saline maintains a stable trend, whereas the dissolution is significantly more pronounced in RPMI and RPMI with PBS.

**Table 2:** Lifetime Evolution of Onivyde in RPMI and RPMI with FBS^a^

time	lifetime in RPMI [ns]	lifetime in RPMI + FBS [ns]
5 min	5.44 ± 0.11	4.80 ± 0.08
30 min	4.59 ± 0.07	4.39 ± 0.03
60 min	4.37 ± 0.06	4.11 ± 0.04
120 min	4.09 ± 0.04	3.98 ± 0.04
6 h	4.03 ± 0.05	3.85 ± 0.04

a The table displays the mean and standard deviation of the fluorescence lifetime of Onivyde in both RPMI and RPMI with FBS. The data show a faster evolution toward shorter lifetimes in the presence of FBS.

FLIM data do not clarify whether the irinotecan molecules released over time are retained within the aqueous lumen of liposomes or leak out into solution over time. To tackle this issue, we measured Onivyde fluorescence while increasing the concentration of potassium iodide (KI), an effective membrane-impermeable dynamic quencher of fluorophores.^29,30^ Based on the data already reported in Figure 4A (and on the overlap of lifetime phasors measured at 2 and 6 h in RPMI, see Figure S4), an incubation time of Onivyde in KI of 2 h was selected. As reported in Figure 4B, the nonplateauing KI quenching effect observed in RPMI is a clear indication of extensive irinotecan leakage into the solution.

To validate this result, we performed standard HPLC-MS release analysis of the solution containing Onivyde liposomes over time. In keeping with FLIM, HPLC-MS analysis detected the presence of an increasing amount of irinotecan molecules over time in the RPMI solution (but not in saline solution; Figure 4C). SN-38 (LOD = 0.069 μM) was not detected during the experiment, allowing us to transcend a possible degradation of the irinotecan into SN-38. Fitting the HPLC data to a monoexponential decay model (*R*^2^ > 0.92) yielded an estimate of irinotecan release kinetics in the two tested solutions: irinotecan leaks out of the liposomes much faster in RPMI (*Τ*_RPMI_ = 1.89 h) than in saline solution (*Τ*_saline_ = 280.01 h), as expected. Indeed, FLIM is sensitive to the total amount of free irinotecan in the system, i.e., the sum of irinotecan molecules outside and those inside the liposomes; by contrast, HPLC only detects irinotecan molecules outside the liposomes.

The HPLC-derived molar fraction of free irinotecan outside of liposomes can be combined with eqs 7.a–7.c to derive an independent estimate of the characteristic lifetime of membrane-associated irinotecan species, which resulted to be 6.05 ± 0.12 ns, in good agreement with the value inferred by means of the heating experiment discussed previously (Figure 2D values are reported in Table 1).

### FLIM Signature of Onivyde in the Cellular Environment

**Figure 5:**
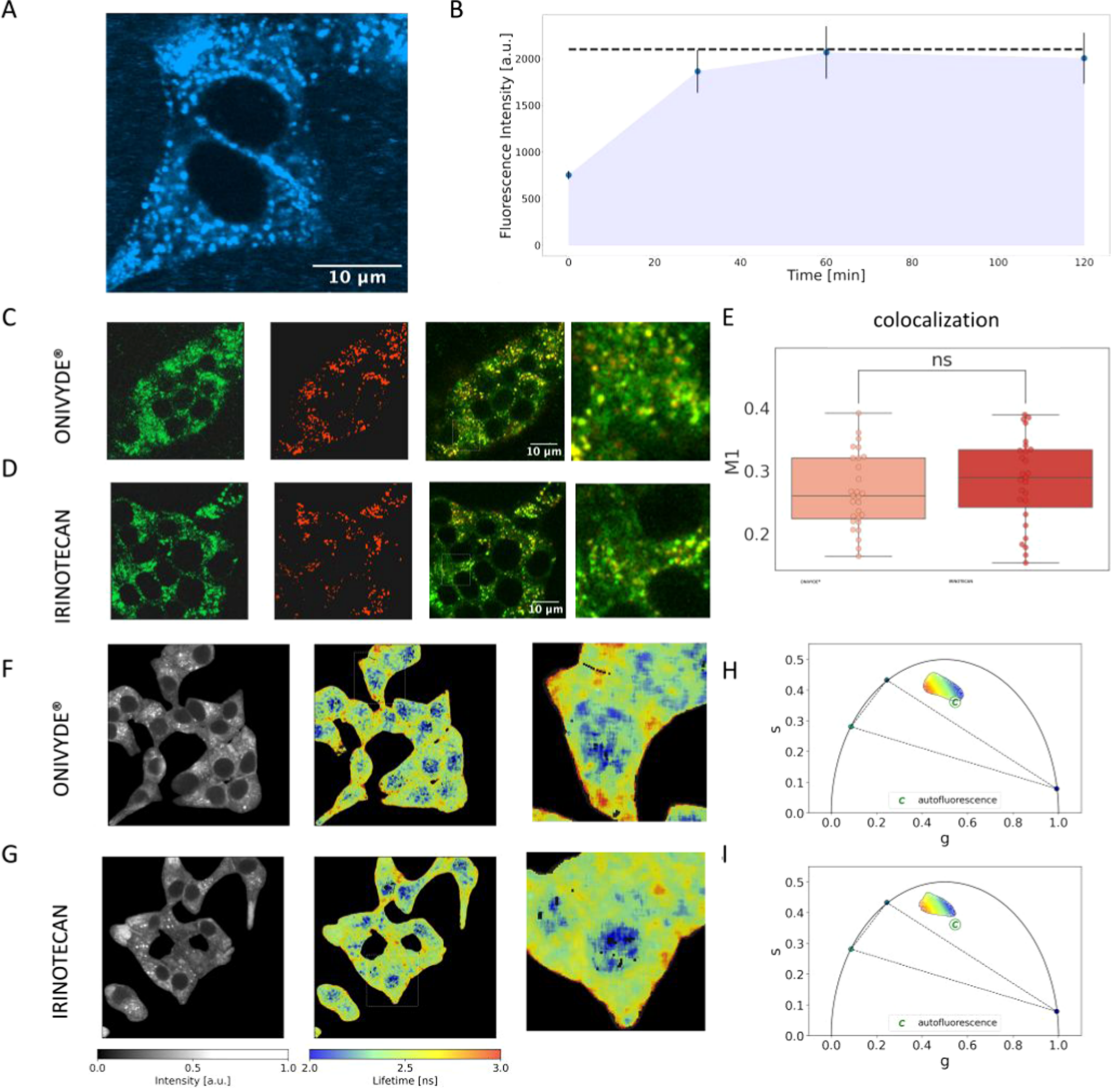
Cellular uptake of Onivyde: confocal and FLIM analyses. (A) Characteristic confocal image of a INS-1E cell exposed for 60 min to Onivyde: both diffuse (mainly cytoplasmic) and punctuate signals can be recognized. (B) Overall cellular uptake of the drug was monitored in terms of average fluorescence intensity within cells in time. (C) Onivyde within INS-1E cells (in green, left panel), Lysotracker staining of the same cells (in red, middle panel), and overlay of the two signals (right panel + zoom). (D) Irinotecan within INS-1E cells (in green, left panel), Lysotracker staining of the same cells (in red, middle panel), and overlay of the two signals (right panel + zoom). (E) The *M*_1_ Manders coefficient indicates marked drug localization within lysosomes for both Onivyde (dark red) and irinotecan (light red) (*N* = 3). (**F**) FLIM analysis of Onivyde within INS-1E cells: intensity (left panel, gray scale) and lifetime (middle and right panels, color-coded) images. (G) FLIM analysis of irinotecan within INS-1E cells: intensity (left panel, gray scale) and lifetime (middle and right panels, color-coded) images. **(H, I**) Characteristic phasor-FLIM signatures of Onivyde and irinotecan in cell with respect to the reference triangle of the three pure species and to cell autofluorescence (labeled as ′′C′′).

At this point, the intrinsic fluorescence of irinotecan was used to monitor the Onivyde uptake in living cells (Figure 5A). Liposomes were administered to living INS-1E cells (rat-derived model of pancreatic β-cells) and confocal imaging performed to monitor cell internalization over time. As shown in the plot of Figure 5B, the intracellular signal rapidly increases, reaching a plateau approx. 1 h after Onivyde administration in the medium. Inspection of confocal images reveals a marked prevalence of the cytoplasmic signal, both diffuse and punctuate, over the nuclear one. A dual-channel colocalization assay using Lysotracker Deep Red in Figure 5C,D demonstrated that the punctuate fluorescence pattern corresponds to the irinotecan signal trapped within acidic subcellular vesicles (i.e., lysosomes). Based on drug-leakage data, it can be inferred that cells treated with Onivyde are simultaneously exposed to encapsulated irinotecan and to a fraction of free-in-solution prodrug, rapidly increasing over time (due to its leakage from liposomes). To further probe the extent of the contribution of the free prodrug to the uptake process, we performed a control experiment using nonencapsulated irinotecan. As reported in Figure 5E, irinotecan yields a similar intracellular fluorescence pattern with respect to Onivyde: diffuse and punctuate cytoplasmic signals, with this latter corresponding to acidic organelles (Figure 5F,G). The similarity extends to the phasor-FLIM signatures observed in cells for the two compounds, as reported in Figure 5H,I (see also the two-sample Kolmogorov–Smirnov test in Figure S5). Worthy of note, the FLIM signatures of both Onivyde and free irinotecan in cells fall outside of the interpretative framework established in-cuvette. It can be envisioned that the intracellular environment contributes to the observed shift toward shorter average lifetimes either by adding its own autofluorescence lifetime (indicated in the phasor plot by the ′′C′′ label) and by driving processing of the internalized irinotecan into metabolites (e.g., by enzymatic conversion) outside of the interpretative triangle.

**Figure 6:**
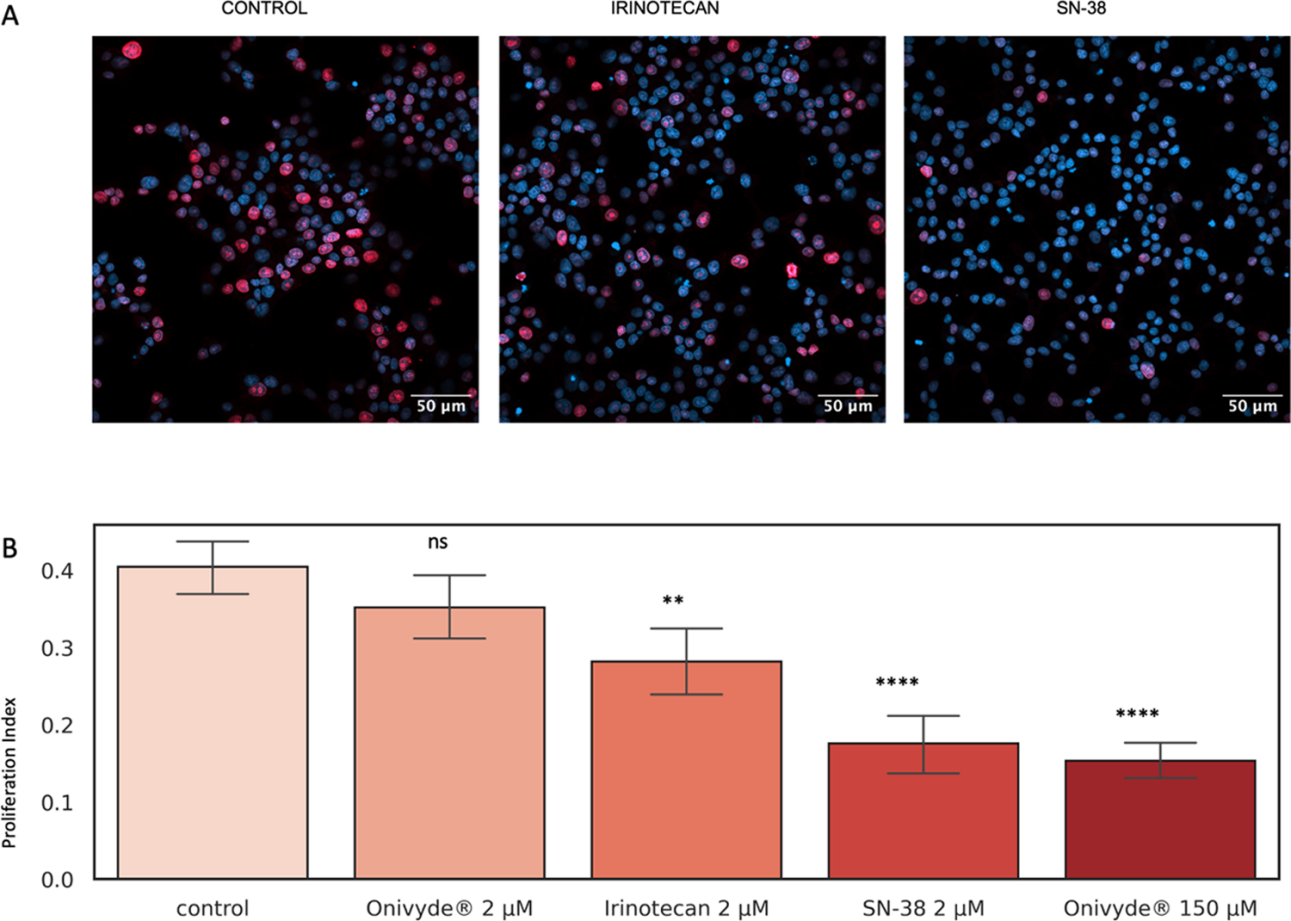
Ki67 proliferation assay in INS-1E cells. (A) Exemplary images for control, irinotecan, and SN-38 treatments (left to right). (B) Proliferation index of different irinotecan formulations compared to control, plotted with 95% confidence intervals. Statistical significance of the proliferation index difference with respect to control was assessed via the Mann–Whitney–Wilcoxon test (*N* = 3) two-sided with Bonferroni correction in 3 independent replicas. Please note that since both irinotecan and SN-38 have low solubility in water and require prior dissolution in DMSO at physiological pH, we minimized the DMSO effect on cells by diluting 1 mM stocks achieving 2 μM solutions, while control cells received the same amount of DMSO. Onivyde at high concentration was given at a dosage of approx. 150 μM, adhering to the manufacturer’s recommended drug-dilution concentration.

To assess whether the similarities observed so far between encapsulated and nonencapsulated irinotecan translated into a similar functional effect on cell proliferative activity, we used the Ki67 assay, a clinical standard for evaluating tumor proliferation.^31−34^ The ratio of red-labeled cell nuclei (red signal indicates ongoing replication) over the total (blue-labeled nuclei) is used as the “proliferation index” to estimate the treatment effect (Figure 6A, see also the Materials and Methods section for further details). Figure 6B shows the results obtained for different formulations compared to control (untreated) cells.

In the experimental conditions used, 2 μM of nonencapsulated irinotecan administered in solution was enough to induce a statistically significant effect on cell proliferation (Figure 6B). By contrast, the same amount of encapsulated irinotecan did not significantly affect the cell proliferation. Based on what is observed so far, this result is not entirely unexpected: while addressing irinotecan poor solubility in water, in fact, encapsulation certainly limits prodrug bioavailability for enzymatic conversion into SN-38. In line with these considerations, 2 μM of nonencapsulated SN-38 produced a more pronounced effect on cell proliferation than both encapsulated and nonencapsulated irinotecan. Worthy of note, the concentration of encapsulated irinotecan must be markedly increased (up to 150 μM) to match the performance of 2 μM of the active drug.

### FLIM Unveils the Signature of SN-38 Release into the Cellular Environment

**Figure 7:**
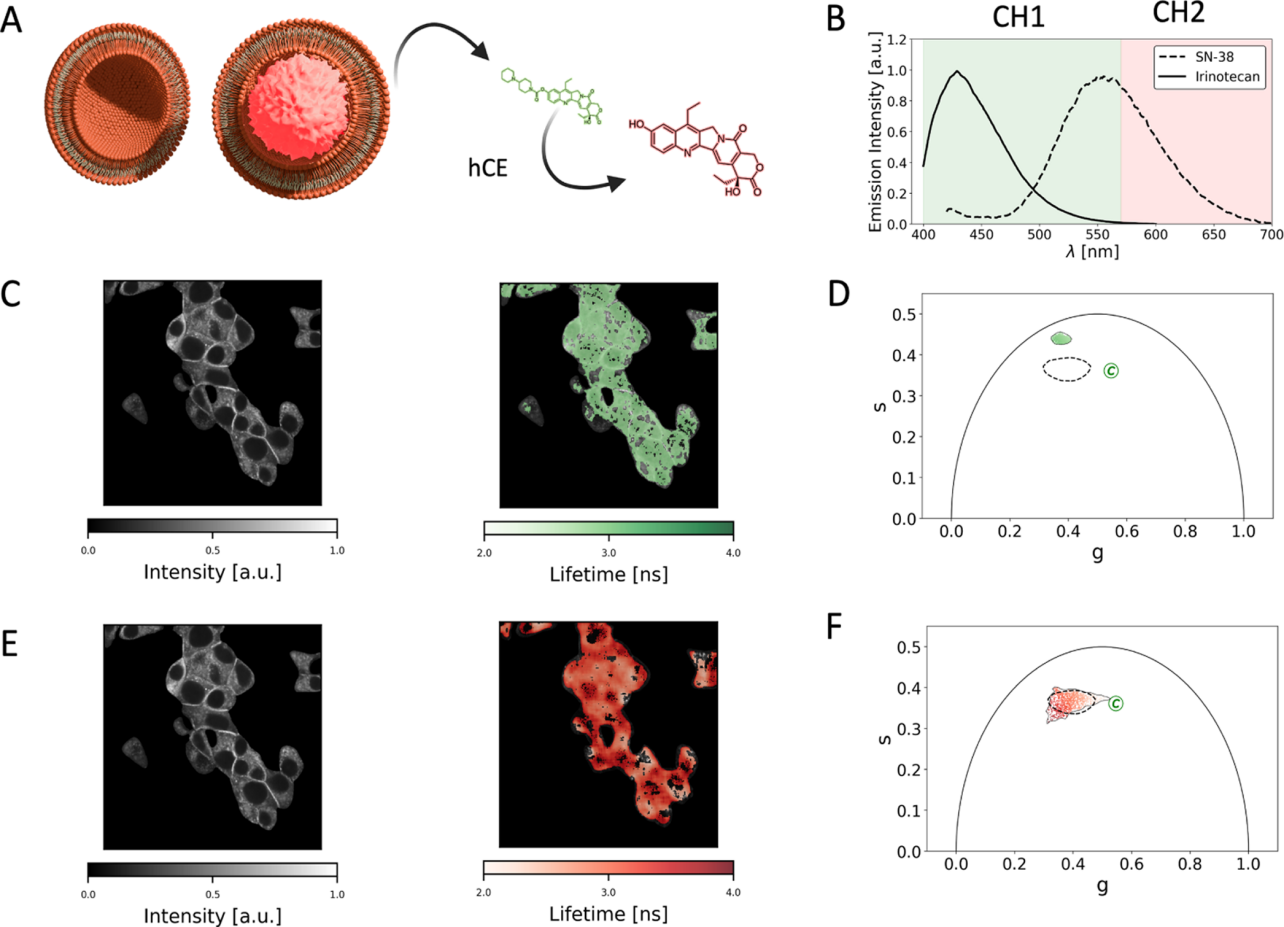
FLIM 2-channel analysis unveils SN-38 presence in INS-1E cells. (A) In the cellular environment, irinotecan is converted to a minor extent into the SN-38 metabolite (highlighted in red) by enzymatic cleavage. (B) SN-38 is characterized by a red shift in emission with respect to irinotecan. (C) Characteristic fluorescence-intensity and lifetime images of INS-1E cells exposed to SN-38 in the green channel (380–570 nm). (D) Onivyde characteristic phasor-FLIM signature in the green channel (380–570 nm) is significantly different from the phasor-FLIM signature of SN-38 (extrapolated from Figure S6D). (E) Characteristic fluorescence-intensity and lifetime images of INS-1E cells exposed to SN-38 in the red channel (above 570 nm). (F) Onivyde characteristic phasor-FLIM signature in the red channel (above 570 nm) is significantly superimposed to that of SN-38 (extrapolated Figure S6D).

The effect of both encapsulated and nonencapsulated irinotecan on cell proliferation must rely on the enzymatic cleavage of the prodrug and release of SN-38 (Figure 7A). As illustrated in Figure 7B, SN-38 exhibits a distinctive red-shifted fluorescence emission compared to irinotecan. Please note that, similarly to irinotecan, SN-38 enters the cells and distributes throughout the cytoplasm with detectable entrapment within punctuate structures (presumably lysosomes) as shown in Figure S6. By exploiting the red shift, SN-38 intracellular distribution and characteristic FLIM signature were defined by exciting at 760 nm and collecting fluorescence above 570 nm.

Using SN-38 phasor-FLIM signature as a reference, we set out a 2-channel FLIM experiment to discriminate the lifetime signatures of irinotecan in Figure 7**C,D** (“green” channel in the 380–570 nm range) and SN-38 in Figure 7E,F (“red” channel in the 570–740 nm range) inside cells exposed to Onivyde. Of note, upon cell segmentation, two distinct phasor clusters were obtained: in the 380–570 nm range, as expected, the FLIM signature of irinotecan is dominating; by contrast, in the 570–740 nm range, a FLIM signature superimposed to the reference of intracellular SN-38 is detected. This is mirrored in the cluster-similarity analysis reported in Figure S 7.

## Conclusions

Despite the significant progress in the development of drug-delivery nanoparticles for clinical applications,^35^ a thorough understanding and control of their complex physicochemical properties remains a challenging task.^36,37^ This largely stems from the lack of analytical tools that can quantitatively dissect the molecular organization of the drug within the formulation throughout the process from manufacturing (synthetic identity)^38,39^ to administration and final fate (biological identity).^28,40^ The resulting lack of knowledge in turn limits our understanding of the performance of nanoencapsulated drugs in current delivery applications and our ability to propose new formulations by rational design. Here, we build on a recently validated FLIM-based approach to describe the nanoscale supramolecular organization of irinotecan in the FDA-approved liposomal formulation Onivyde. We investigated three different experimental conditions of interest: (i) Onivyde in the original manufacturer’s solution, (ii) Onivyde diluted in two relevant solutions, i.e., saline solution for injection and cell-culturing medium (with or without serum proteins), and (iii) Onivyde within the intracellular environment. Concerning point (i), FLIM unveiled that irinotecan coexists in two distinct physical states within the Onivyde formulation: gelated/precipitated irinotecan and membrane-associated irinotecan, the latter being not deducible from the manufacturer’s indications. Thus, FLIM analysis unveiled the absence of free-in-solution irinotecan in the manufacturer’s formulation, a result not entirely surprising based on the poor water solubility of irinotecan. Concerning point (ii), FLIM allowed monitoring irinotecan leakage from liposomes upon Onivyde dilution. We demonstrated that the gelated/precipitated state of irinotecan rapidly transforms (within 2 h) into the free prodrug, which in turn leaks out of the liposome. FLIM-based indications were validated by HPLC. The similarity observed between encapsulated and nonencapsulated irinotecan in-cuvette mirrored their behavior in cells (point (iii)). Indeed, confocal imaging and cell-proliferation assays yield similar results for encapsulated and nonencapsulated irinotecan in terms of both cell-uptake mechanism and cell-division inhibition. Finally, the implementation of 2-channel FLIM analysis enabled the discrimination of irinotecan and SN-38 intracellular signatures upon delivery of the encapsulated drug. This analysis demonstrated the presence of SN-38 in cells exposed to Onivyde. In conclusion, we believe that the present approach has the potential to complement standard methods for investigating how the synthetic identity of a drug may be altered at any level from manufacturing to final fate within the intracellular environment. Achieving control over the physicochemical properties of nanocarriers yields the potential to boost both manufacturing processes and drug-delivery design.

## Supporting Information Available

Investigation for second harmonic generation; schematic representation of the graphical approach for assessing intensity fractions of coexisting species; validation of the protocols used with gelated/precipitated irinotecan; onivyde lifetime evolution in cellular media, Kolmogorov–Smirnov (K–S) tests ran on treatments of INS-1E cells with different nanoformulations; and FLIM characterization of SN-38 in cells (PDF)
