# Supplementary material for: Fluorescence Lifetime Nanoscopy of Liposomal Irinotecan Onivyde: From Manufacturing to Intracellular Processing
Source: ACS Appl Bio Mater. 2023 Sep 12;6(10):4277–89. doi: 10.1021/acsabm.3c00478 (PMC10583229; doi:10.1021/acsabm.3c00478)
Supplement: Supplementary file 1 — mt3c00478_si_001.pdf [file mt3c00478_si_001.pdf]

SUPPORTING INFORMATION

Fluorescence lifetime nanoscopy of liposomal irinotecan Onivyde®: from manufacturing to intracellular processing

Authors:

Mario Bernardi[a], Giovanni Signore[b,c], Aldo Moscardini[a], Licia Anna Pugliese[a], Luca Pesce[a], Fabio Beltram[a, d], Francesco Cardarelli[a, d, *]

Affiliations:

[a] Scuola Normale Superiore, Laboratorio NEST, Piazza San Silvestro 12, 56127 Pisa, Italy

[b] Biochemistry Unit, Department of Biology, University of Pisa, via San Zeno 51, 56123 Pisa, Italy

[c]Institute of Clinical Physiology, National Research Council, 56124 Pisa, Italy.

[d] NEST, Istituto Nanoscienze-CNR, Piazza S. Silvestro, 12, I-56127, Pisa, Italy

[*]To whom correspondence should be addressed: francesco.cardarelli@sns.it

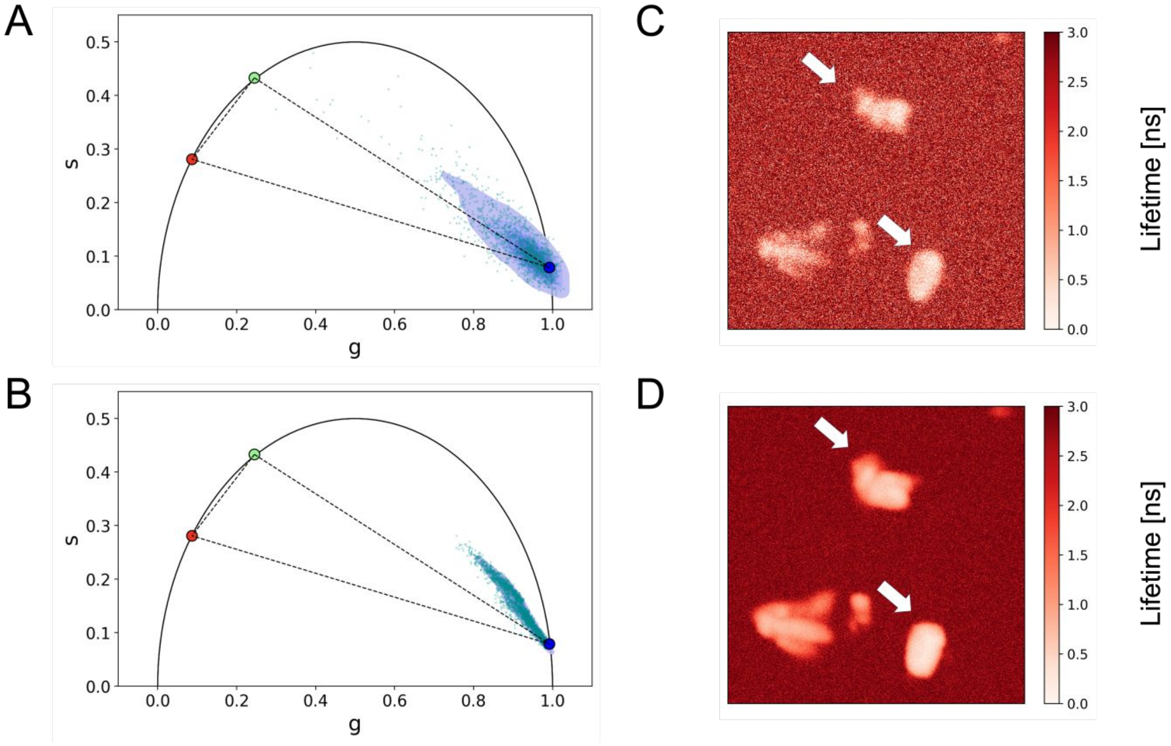

Figure S 1 Investigation for Second Harmonic Generation A) Lifetime raw data of gelated/precipitated irinotecan in the range 380-470 nm. **B**) Lifetime raw data of precipitate irinotecan in the range 490-530 nm. **C**) Irinotecan precipitate lifetime image captured in the 380-470-nm range. **D**) Gelated/precipitated irinotecan lifetime image captured in the 490-530-nm range. These results provide insights into the behavior of irinotecan precipitates in different spectral ranges and support our findings on the absence of Second Harmonic Generation in the study of these compounds.

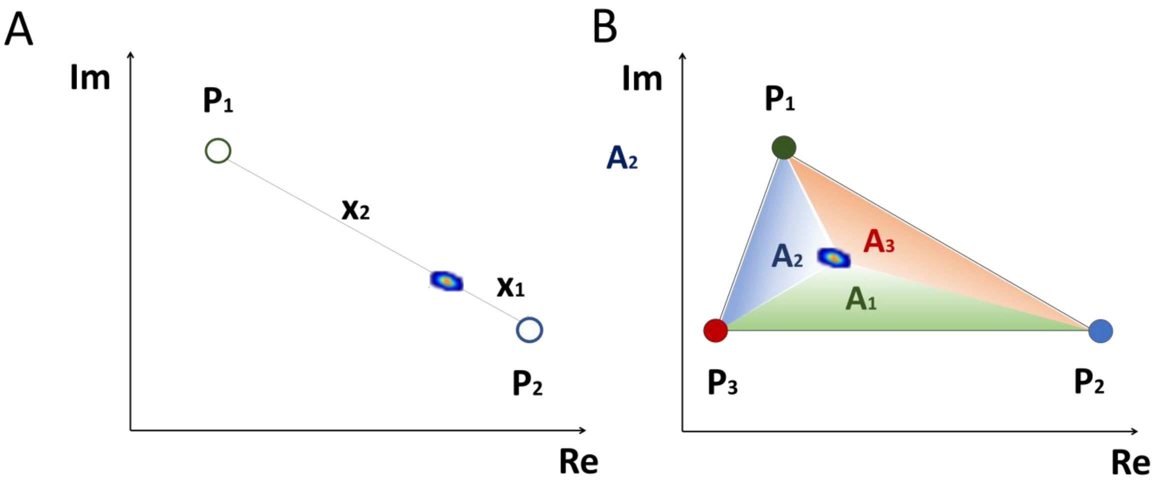

Figure S 2 Schematic representation of the graphical approach for assessing intensity fractions of coexisting species. **A**) Linear approach for 2 coexisting species. **B**) Approach for 3 coexisting species.

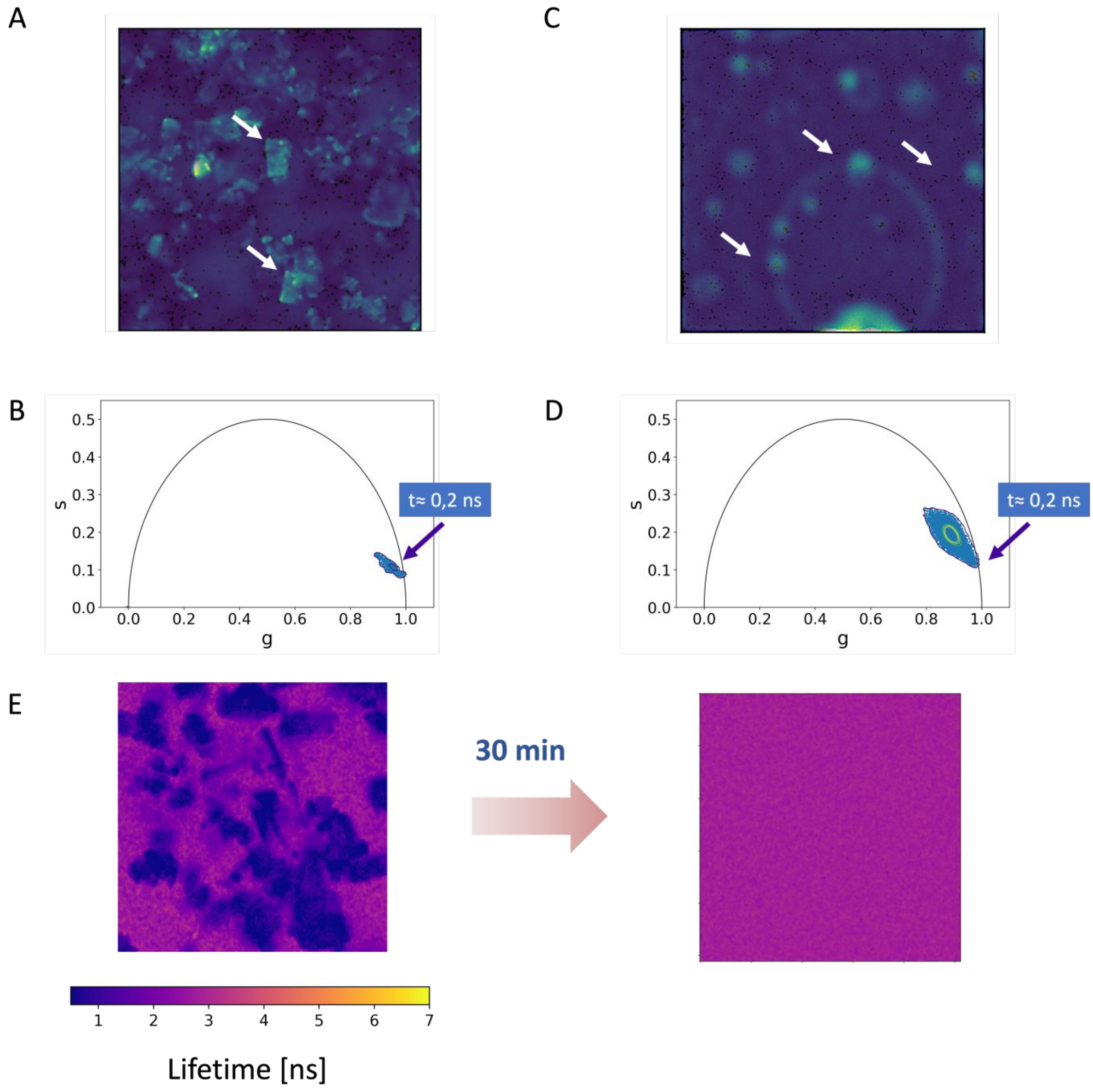

Figure S 3 Validation of the protocols used with gelated/precipitated irinotecan A) Fluorescence intensity image of gelated/precipitated irinotecan, indicated by the arrow. **B)** Lifetime raw data of gelated/precipitated irinotecan in the phasor plot. **C)** Fluorescence intensity image of Onivyde® after spin-coating on the glass at 5000 rpm for 1 min; arrows indicate residuals of the putative gelated/precipitated/gel form of irinotecan. **D)** Lifetime raw data of gelated/precipitated clusters from Onivyde® after the spinning experiment are in agreement with the outcome of the precipitation protocol. **E)** Heating effect on gelated/precipitated irinotecan after 30 min at 90°C.

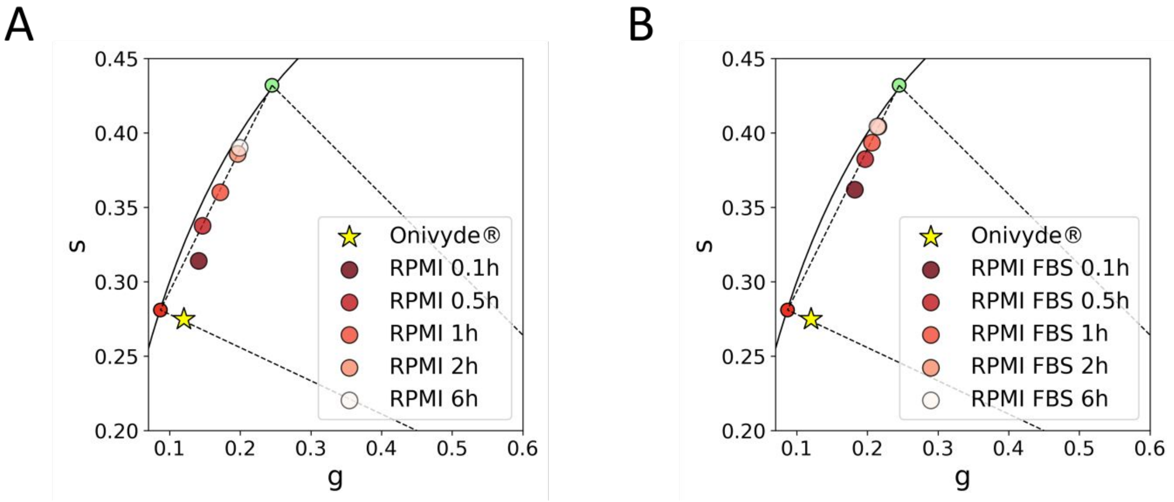

Figure S4 Onivyde® lifetime evolution in cellular media A) Onivyde® lifetime evolution in RPMI medium is completed in 2h as demonstrated by the overlap between the signal at 120 min and 6h, Onivyde® starting phasor is represented with a star. **B)** Onivyde® evolution in FBS-enriched medium represented on the phasor plot.

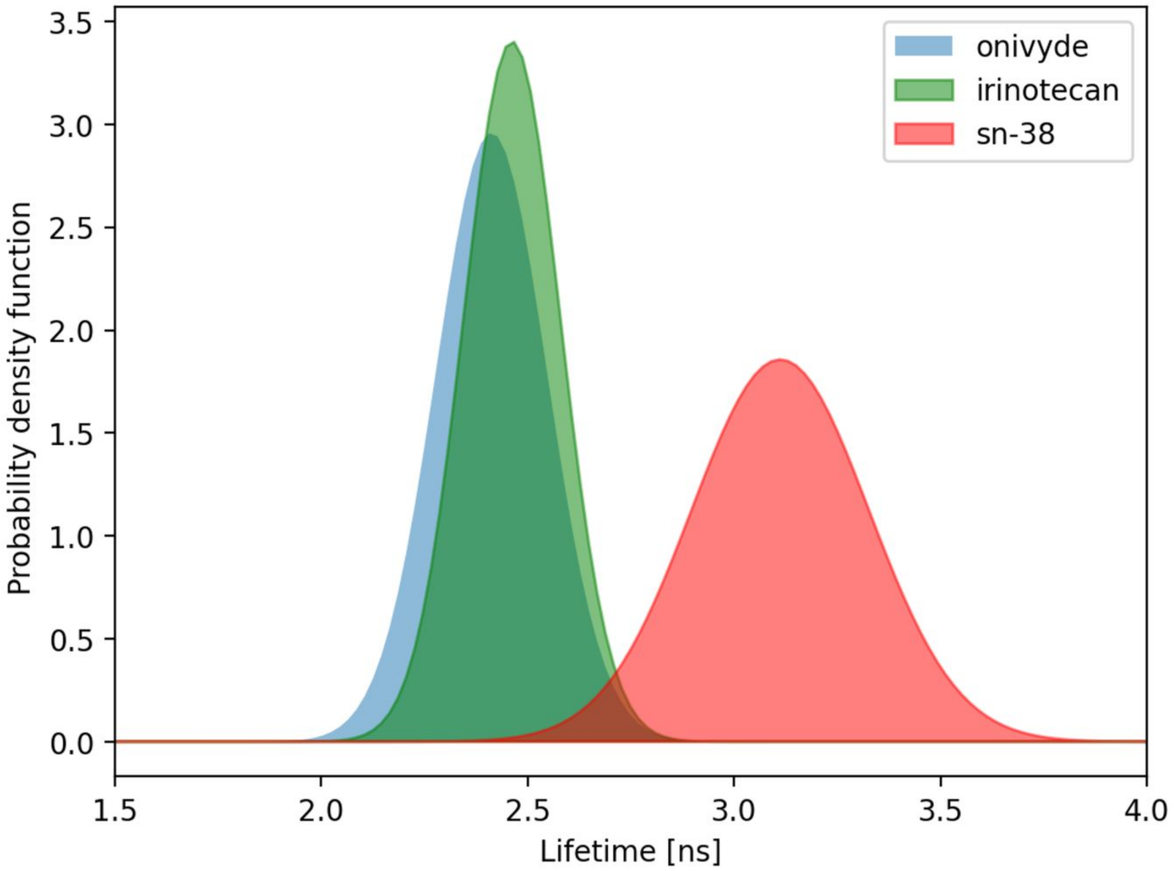

**Samples**	Onivyde	Irinotecan	SN-38
Onivyde	1	0.81	6.28 10^{-5}
Irinotecan	0.81	1	3.75 10^{-5}
SN-38	6.28 10^{-5}	3.75 10^{-5}	1

data are consistent with equality null

data are inconsistent with equality null

Figure S5 Kolmogorov Smirnov (K-S) test ran on treatments of INS-1 E cells with different nanoformulatio*ns.* K-S test of irinotecan, SN-38 and Onivyde® lifetime distribution.

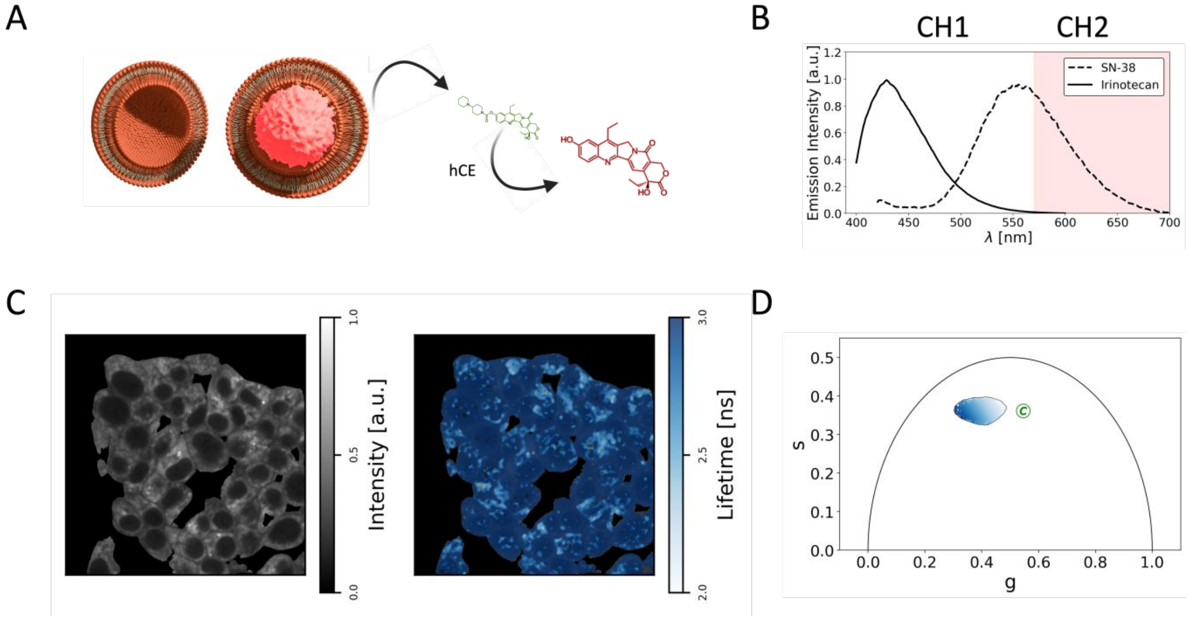

Figure S6 FLIM characterization of SN-38 in cells A) In the cellular environment irinotecan is converted to a minor extent into the SN-38 metabolite (highlighted in red) by enzymatic cleavage **B)** SN-38 exhibits a notable emission red shift compared to irinotecan, emitting above the 380-570 nm range, as highlighted in red **C)** Characteristic fluorescence-intensity and lifetime image of INS-1E cells exposed to SN-38 **D)** Characteristic phasor-FLIM signature of cells exposed to SN-38.

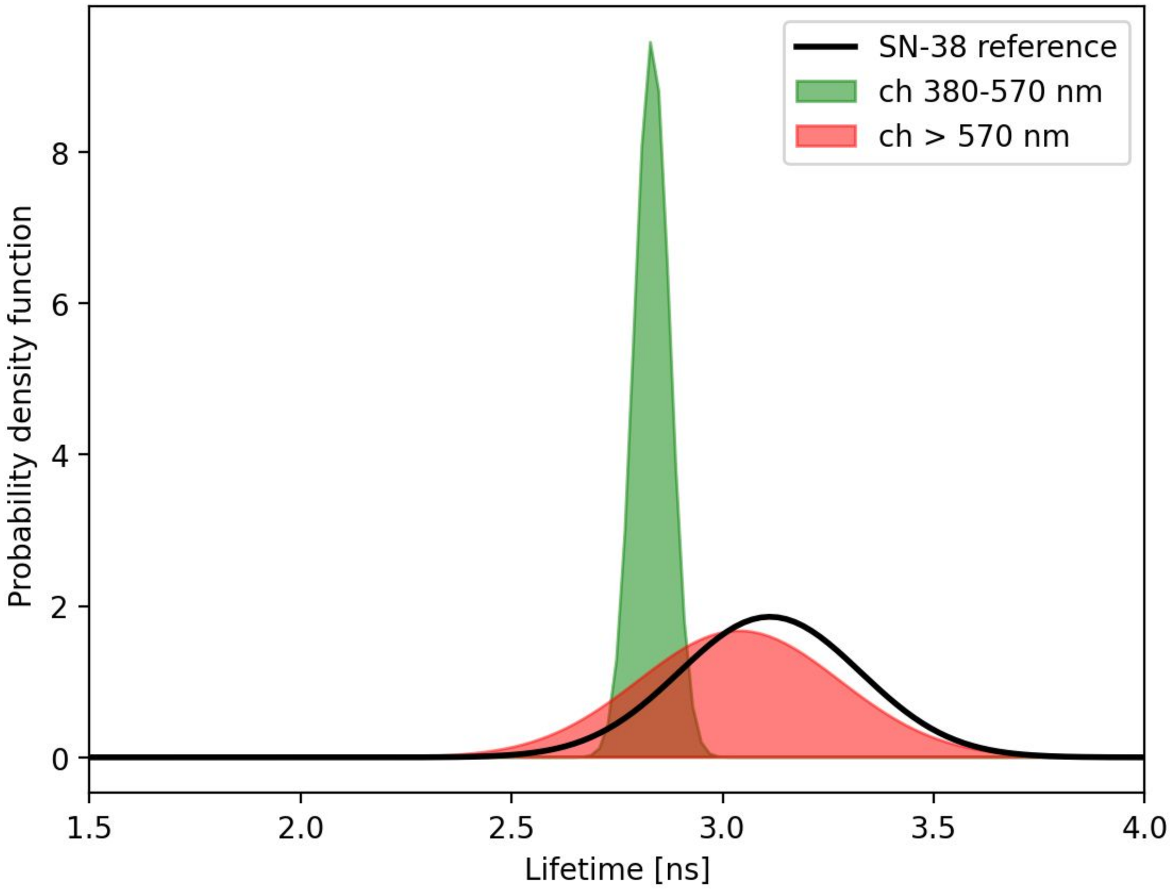

**Samples**	SN-38	Red channel	Green channel
SN-38	1	0.27	$2.87\ 10^{-62}$
Red channel	0.27	1	$4.78\ 10^{-62}$
Green channel	$2.87\ 10^{-62}$	$4.78\ 10^{-62}$	1

data are consistent with equality null

data are inconsistent with equality null

Figure S 7 Kolmogorov Smirnov (K-S) test ran on treatments of INS-1 E in two different channels. K-S test of SN-38 and Onivyde® (in the green and red channel) lifetime distribution.
